# Fecal microbiota transplantation in hematopoietic cell transplant and cellular therapy recipients: lessons learned and the path forward

**DOI:** 10.1080/19490976.2023.2229567

**Published:** 2023-06-29

**Authors:** Shaghayegh Habibi, Armin Rashidi

**Affiliations:** a Lucy Curci Cancer Center, Eisenhower Health, Rancho Mirage, CA, USA; b Clinical Research Division, Fred Hutchinson Cancer Center; and Division of Oncology, University of Washington, Seattle, WA, USA

**Keywords:** Cellular therapy, Dysbiosis, Fecal microbiota transplantation, Graft-versus-host disease, Hematopoietic cell transplantation, Microbiota

## Abstract

Disruptions to the gut microbiota have been associated with adverse outcomes including graft-versus-host disease, infections, and mortality after hematopoietic cell transplantation and cellular therapy. Evidence for causal links is accumulating, thus supporting therapeutic interventions targeting the microbiota with the goal of preventing and treating adverse outcomes. One such intervention is fecal microbiota transplantation (FMT) by which an entire community of gut microbiota is transferred to the patient with dysbiosis. As this approach in transplant and cellular therapy recipients is still in its infancy, no best approach has been defined and many open questions need to be addressed before FMT becomes a standard treatment. In this review, we highlight microbiota-outcome associations with the highest level of evidence, provide an overview of the main FMT trials, and suggest some paths forward.

## Introduction

### Critical role of the gut microbiota in tissue and organismal homeostasis

Harboring 10^13^ to 10^14^ microbial organisms, the gut represents the most heavily colonized organ in the human body.^1^ This community of microbes, referred to as the gut microbiota, plays numerous roles in host physiology. To name a few, the gut microbiota is critical in (i) synthesis of essential amino acids and vitamins, and metabolism of indigestible dietary polysaccharides,^2^ (ii) colonization resistance, the process by which the gut microbiota resists invasion by other microorganisms, thus protecting the host from infections,^3^ (iv) maintenance of a robust intestinal barrier and its repair after injury,^4,5^ (v) regulation of intestinal and systemic immunity,^6,7^ and (vi) production of many metabolites that circulate in the bloodstream, with distant and organism-level physiological effects.^8^ Not surprisingly, disruptions to the gut microbiota have been implicated in common diseases such as obesity,^9^ depression,^10^ and type 2 diabetes.^11^ Recent evidence for the presence of axes such as the gut-brain axis,^12^ gut-lung axis,^13^ and even brain-gut-kidney axis^14^ attest to the broad involvement of the gut microbiota in tissue and organismal homeostasis.

### Hematopoietic cell transplantation and chimeric antigen receptor T-cell therapy

Hematopoietic cell transplantation (HCT) and chimeric antigen receptor (CAR) T-cells are potentially curative treatments for patients with hematologic malignancies. The HCT process starts with administration of a conditioning regimen consisting of chemotherapy with or without total body irradiation, followed by infusion of hematopoietic cells. Hematopoietic cells may be derived from the same patient (autologous) or another individual (allogeneic). Following the infusion of the graft, there is a period of profound immunosuppression lasting weeks to months, during which patients receive antibiotics to prevent and treat infections. Enteral nutrition is often compromised due to the toxicity of conditioning regimens to the upper and lower intestinal tract. Infections and graft-versus-host disease (GVHD; acute or chronic) are common complications of HCT.

Acute GVHD is the clinical outcome of an early alloimmune attack by donor T cells against host tissues. In intestinal acute GVHD, intestinal stem cells seem to be the main target in this attack.^15^ Detailed reviews of the classic pathophysiology of acute GVHD have been published^16^ and more recent translational aspects of this process have been highlighted.^17^ A critical pathway in acute GVHD pathogenesis involves the effects of the conditioning regimen (esp. high-dose total body irradiation) used before HCT on the intestinal microenvironment. Conditioning regimens damage the intestinal barrier separating the intestinal microbiota from subepithelial immune cells. Intestinal tissue injury leads to the recruitment and activation of neutrophils, triggering acute GVHD. Tissue damage may occur directly by activated neutrophils.^18^ In addition, activated neutrophils can migrate to mesenteric lymph nodes, a process dependent on the intestinal microbiota. Once in the mesenteric lymph nodes, these neutrophils present antigens to T cells via MHC class II and lead to their activation.^19^ Conditioning also augments expression of microbiota-derived Toll-like receptor ligands and MHC class II on intestinal epithelial cells, triggering downstream pro-inflammatory pathways via macrophages, innate lymphoid type 1 cells, and conventional T cells.^20^ The intestinal microbiota regulates intestinal tissue sensitivity to chemotherapy and radiation.^4,5^

CAR-T cells are a more recent immunotherapeutic treatment modality for patients with specific lymphoid malignancies.^21^ CARs are synthetic receptors expressed by an immune effector cell, typically T cells, which recognize cognate antigens on target cancer cells. Before CAR T-cell infusion, low-dose lymphodepletion chemotherapy is administered. The consequent immunosuppression triggers the use of prophylactic and therapeutic antibiotics. Infection, cytokine release syndrome (CRS), and immune effector cell – associated neurotoxicity syndrome (ICANS) are common complications of CAR-T cell therapy.

In this review, we first discuss gut microbiota disruptions in HCT and CAR-T recipients and the potential clinical impact of these disruptions. Next, we focus on one of the strategies – fecal microbiota transplantation (FMT) – to repair microbiota injuries and improve clinical outcomes in these patients. We synthesize the strongest pieces of evidence from the rapidly expanding literature.

## Gut microbiota disruptions in HCT and CAR-T therapy

The two most consistent features of dysbiosis repeatedly observed in HCT recipients are loss of alpha diversity and pathobiont overgrowth. Alpha diversity is measured by various indices of microbial richness (number of different taxa) and evenness (taxa relative abundances).^22^ As HCT recipients frequently have a history of treatment for their underlying blood disorder, the process of dysbiosis may have already started by the time they are referred for HCT. As a result, when they arrive for HCT, the gut microbiota may show a “scar” of prior injuries.^23–25^ In addition, after allo-HCT, the gut microbiota diversity further declines.^23,26–28^ Diversity loss is commonly associated with decreased abundance of obligate anaerobic commensals^29,30^ and domination by a few species. Intestinal domination has been most consistently demonstrated for *Enterococcus*.^31–34^

After the removal of various causes of gut microbiota injury, partial though slow resolution of damage seems to occur.^27,35^ However, published data on long-term trajectory of microbiota after HCT are limited. In the longest follow-up (median >6 years) thus far of the gut microbiota in allogeneic HCT recipients (59 patients), gut microbial diversity and the abundance of butyrate-producing bacteria continued to be lower than the age- and sex-matched healthy control,^36^ supporting the microbiota scar theory.

## Causes of gut dysbiosis in HCT and CAR-T therapy

Antibiotic exposure is arguably the leading cause of dysbiosis in HCT recipients. Piperacillin-tazobactam and carbapenems are the antibiotics most consistently associated with microbiota injury in HCT patients.^37–43^ Potent activity against obligate anaerobic bacteria is a shared feature of these antibiotics. In murine studies, carbapenem use led to the expansion of mucus-degrading species *Akkermansia muciniphila*^38^ and *Bacteroides thetaiotaomicron*.^37^ The use of anti-anaerobic antibiotics is also associated with *Enterococcus* expansion.^33^

Conditioning regimens^44^ and nutrition (enteral vs. parenteral, calorie-rich vs. calorie-restricted)^45–47^ may influence the gut microbiota in HCT patients. Enteral nutrition is possibly beneficial for clinical outcomes and may improve microbiota indices but the evidence is weak and more rigorous trials are needed.^48^ In a large study of allogeneic HCT patients combined with mechanistic murine studies, lactose drove *Enterococcus* expansion in the gut which was associated with lower overall survival and higher acute GVHD-related mortality.^32^

The gut microbiota is modulated by several other intrinsic and extrinsic factors. Although evidence in the setting of HCT and CAR-T therapy is limited,^49^ large population-based studies have found relations between the gut microbiota and variables including anthropometric measures (age, sex, body mass index), stool consistency, common diseases (e.g. irritable bowel syndrome, coronary heart disease, depression, anemia), non-antibiotic drugs (e.g. proton pump inhibitors, statins, laxatives, opioids, antiemetics), and smoking history (direct or indirect exposure).^50–53^ Some of these variables are frequently present or relevant to HCT and cellular therapy recipients.

## Gut dysbiosis and outcomes of HCT and CAR-T therapy

Associations have been reported between dysbiosis and various outcomes of HCT and CAR-T therapy. However, the most consistent associations have been for acute GVHD and transplant-related mortality (TRM; mortality of any cause other than relapse of the underlying malignancy).

### Acute GVHD

Dysbiosis has been associated with more GVHD in several studies.^28,54–58^ Microbiota changes associated with acute GVHD or its mortality include loss of alpha diversity,^28^ loss of butyrogenic bacteria,^54,58^ loss of Clostridia,^58^ loss of *Blautia*,^28,59^ loss of Lachnospiraceae,^55^ lower ratios of strict-to-facultative anaerobic bacteria,^58^ colonization with oral Actinobacteria and Firmicutes,^55^ expansion of picobirnaviruses,^56^ *Enterococcus* domination,^32^ and large fluctuations in microbial community composition (“microbial chaos”) in the early phase after HCT.^57^ Certain microbiome-associated systemic metabolomic alterations can be detected at the onset of acute GVHD. In one analysis, the concentration of several microbiota-derived metabolites (aryl hydrocarbon receptor ligands, bile acids, and plasmalogens) at the onset of acute GVHD was significantly different from healthy donors.^60^

We recently analyzed a large single-center database of allogeneic HCT recipients (>2,000 patients between 2010–2021).^61^ A total of 94 variables including 17 antibacterial antibiotic classes used between 7 days before and 30 days after transplant (weekly intervals) along with relevant clinical and demographic variables were analyzed. We applied three orthogonal statistical methods: conventional Cox proportional hazard regression, marginal structural models, and machine learning. Marginal structural models consider antibiotic exposures on a given day as a function of antibiotic exposures on the previous day and the baseline covariates. By machine learning, we attempted to reveal non-linear, more complex associations between antibiotic use and acute GVHD. Exposure to carbapenems during weeks 1 and 2 after HCT and exposure to combinations of penicillins with a β-lactamase inhibitor (e.g. piperacillin-tazobactam) during week 1 after HCT were most consistently associated with increased risk for acute GVHD. These two classes of antibiotics have been associated with acute GVHD in other studies as well.^37–43^ Intriguingly, the use of metronidazole, another strong anti-anaerobic antibiotic, led to less acute GVHD in a seminal randomized trial.^62^

Preservation of the gut microbiota may be involved or even required in the success of novel therapeutics in prevention of acute GVHD. In a phase 2 trial of tocilizumab (an interleukin-6 inhibitor) used for GVHD prophylaxis, the expected loss of microbiota diversity after HCT was less than in a historical control cohort. Similarly, *Enterococcus* domination was less frequent among patients receiving tocilizumab than historical controls.^63^ In the therapeutic setting, a phase 2 trial of a novel recombinant human interleukin-22 dimer used in combination with systemic corticosteroids for frontline treatment of lower gastrointestinal acute GVHD showed expansion of commensal anaerobes and greater microbiota diversity in responders.^64^

The gut virome has also been associated with the risk of acute GVHD. A viral ‘bloom’ seems to occur after HCT, accompanied with an expansion of vertebrate viral sequences following transplantation. Patients developing gastrointestinal acute GVHD had increased presence and abundance of specific DNA viruses (anelloviruses, herpesviruses, papillomaviruses and polyomaviruses) over time and lower phage richness. Picobirnaviruses were predictive of subsequent intestinal acute GVHD.^56^

### Transplant-related mortality

There is a repeatedly observed association between low gut microbiota diversity and TRM after allogeneic HCT.^23,65^ Acute GVHD and infection are the leading causes of TRM in HCT recipients. A specific timeframe when dysbiosis may impact mortality is weeks 2 and 3 after HCT, coinciding with hematopoietic engraftment.^23,30^ Loss of *Blautia* and expansion of *Enterococcus* have been associated with acute GVHD-related mortality.^32,59^ Loss of urinary 3-indoxyl sulfate and butyrogenic bacteria, associated with reduction of Lachnospiraceae and Ruminococcaceae and expansion of Bacilli, in the first 10 days after allogeneic HCT was predictive of increased TRM.^30,54^

### Other outcomes after HCT

Some data suggest associations between the gut microbiota and bloodstream infection (BSI),^33,66,67^ immune reconstitution,^68,69^ respiratory tract infections,^70,71^ CDI,^72^ chronic GVHD,^73^ and disease relapse^74^ after HCT.

### Outcomes after CAR-T therapy

Microbiota results from CAR-T studies are more recent. In one study, exposure to piperacillin/tazobactam and carbapenems within 4 weeks before CAR-T infusion was associated with worse survival and increased neurotoxicity. Baseline alpha diversity, as well the abundance of *Ruminococcus, Bacteroides, and Faecalibacterium* were associated with complete response to therapy in patients with B-cell malignancies.^75^ These associations were independent of several other variables potentially reflecting more aggressive disease and disease-related complications. Whether exposure to oral vancomycin may have the same association could not be evaluated because exposure to this antibiotic in the absence of piperacillin/tazobactam and carbapenems was infrequent. In another study of B-cell lymphoma patients, piperacillin-tazobactam, meropenem, cefepime or ceftazidime use within 3 weeks before CAR-T infusion was associated with lower response to treatment. Focusing on patients not exposed to these antibiotics, the analysis revealed several bacteria associated with CAR-T response. These bacteria included *Bifidobacterium longum*, *Bacteroides eggerthii, Ruminococcus lactaris, Eubacterium spp. CAG 180, Akkermansia muciniphila*, *Erysipelatoclostridium ramosum*, and *Bacteroides stercoris*.^76^

## Mechanistic evidence

The possible causal link between microbiota and subsequent clinical outcomes is unclear. In the human setting, it is difficult to eliminate confounding with certainty. Conditioning-induced intestinal barrier damage, peak antibiotic exposure, altered nutrition, and maximum microbiota damage tend to occur about the same time after HCT, making it difficult to determine the effect of each one in isolation. As an example, and given the importance of intestinal damage in the pathogenesis of acute intestinal GVHD,^77^ could intestinal damage be the underlying cause for both microbiota injury and acute GVHD without the latter two being causally linked? Mechanistic evidence for causality is accumulating in murine models. However, whether results from controlled murine experiments can be generalized to the human setting is unclear. Germ-free mice, a common and useful model for microbiota studies, do not represent antibiotic-treated patients undergoing HCT as the gut microbiota of patients often contains a large microbial community even after heavy antibiotic exposure. Through coprophagy, specific pathogen-free (SPF) mice may replenish their gut microbiota from their own stool and the stool of their co-housed mice. Finally, a fundamental unanswered question is whether microbiota effects are primarily mediated by individual taxa (e.g. *Enterococcus*, *Akkermansia*) or community-level properties of the microbiota (e.g. diversity). Complicating the matters is the fact that community-level properties such as alpha diversity are frequently associated with the relative abundance of specific taxa. For example, enterococcal domination is very common in low-diversity communities depleted of obligate anaerobic commensals after HCT.^26,32,33^

### Acute GVHD

Loss of short-chain fatty acids (SCFAs; generated from fermentation of dietary fiber) and indole (a tryptophan degradation product) are two metabolic changes that have been repeatedly observed in the setting of dysbiosis in HCT recipients. These microbiota-derived metabolites have immunosuppressive^78–80^ and gut barrier enhancing effects^79–84^ and may protect against acute (indole)^79^ and chronic (butyrate/propionate)^73^ GVHD. Although most results thus far have been in murine studies, cumulative evidence suggests involvement of these metabolites in the outcomes of HCT in patients. In a pilot pharmacomicrobiomics study with 20 adults undergoing allogeneic HCT using mycophenolate mofetil (MMF) and tacrolimus for GVHD prophylaxis, the association between the gut microbiota and enterohepatic recirculation (EHR) of mycophenolic acid was evaluated. Several *Bacteroides* species (*B. vulgatus*, *B. stercoris*, and *B. thetaiotaomicron*) were more abundant in the high vs. low EHR group,^85^ introducing the possibility that the gut microbiota may modulate acute GVHD risk via differential metabolism of prophylactic medications. As MMF is a key drug in GVHD prophylaxis in the increasingly more common HLA-haploidentical HCT, more pharmacomicrobiomics studies are warranted.

Meropenem treatment of mice undergoing allogeneic HCT led to the expansion of *B. thetaiotaomicron* and aggravation of acute colonic GVHD.^37^ Through its mucolytic activity, *B. thetaiotaomicron* caused thinning of the colonic mucus layer and decreased xylose levels within the lumen, a process which was prevented by oral supplementation of xylose. Another study attempted to block the successful entry of live bacteria through the intestinal wall by immunizing mice against the conserved microbial surface poly-N-acetylglucosamine. By depleting bacteria penetrating the gut barrier without altering microbiota diversity, this approach reduced bacterial-induced neutrophil activation and acute GVHD.^86^

In another murine study, *Enterococcus* expansion, a predictor of higher acute GVHD risk in patients, was dependent on lactose. Oral lactose depletion attenuated *Enterococcus* outgrowth and reduced the severity of GVHD, supporting a causal link.^32^ In the opposite direction, the process of immune attack during acute GVHD can cause damage to the gut microbiota by increasing oxygen tension in the normally hypoxic intestinal lumen, leading to a loss of obligate anaerobic commensals. This in turn worsens intestinal pathology. Importantly, oral iron chelation in murine models mitigated dysbiosis and reduced the GVHD severity, supporting a causal link.^87^

### Bloodstream infection

By thinning the intestinal barrier, expanded *A. muciniphila* may increase the translocation of bacteria, their components (e.g. flagellin, lipopolysaccharide), and metabolites to the bloodstream.^46,88,89^ Decreased oral intake potentiates expansion of mucolytic bacteria because they can use mucin as a nutrient.^46,90^ *Enterococcus* expansion in the gut has been repeatedly associated with the risk of enterococcal BSI^33,91^ and Gram-negative bacteria domination of the gut microbiota with Gram-negative bacteremia.^67^ The mechanism seems to be a concentration effect, by which dominant bacteria have a higher chance of passing through the intestinal barrier.

### CAR-T efficacy

Findings from a small retrospective study in patients with B-cell acute lymphoblastic leukemia treated with CAR-T suggested better CAR-T expansion in patients exposed to oral vancomycin before infusion.^92^ In a murine CD19^+^ lymphoma model, microbiota conditioning with vancomycin combined with CD19 CAR-T therapy enhanced cross-presentation of tumor-associated antigens and improved tumor control compared to CAR-T alone.^92^ In another study, peptidoglycan biosynthesis was the metabolic pathway most strongly associated with disease progression after CAR-T, independently of demographic or clinical variables.^76^

## Fecal microbiota transplantation in HCT

HCT recipients experience significant disruptions to their gut microbiota,^93^ with implications for clinical outcomes such as mortality,^23,65^ acute GVHD,^28,37,38,54–58,79^ poor immune reconstitution,^69,94,95^ and disease relapse.^74^ Similar to HCT recipients, specific antibiotic exposures and gut microbiota changes in CAR-T recipients have been associated with treatment response, mortality, and ICANS.^75,76^ Inspired by these associations, strategies to mitigate dysbiosis to improve clinical outcomes after HCT and CAR-T therapy have been a subject of recent interest.^96^ Fecal microbiota transplantation (FMT) is one such strategy by which gut microbial communities are transferred as a whole to the patient to prevent or ameliorate dysbiosis. FMT is currently approved only for the treatment of multiply recurrent or refractory *Clostridioides difficile* infection (CDI).^97^

Restoring the commensal microbiota and eradicating pathogens including multi-drug resistant organisms (MDROs) is the main idea in the prophylactic use of FMT after HCT. Clinical outcomes that might improve with this approach include infection rate, acute (and perhaps chronic) GVHD, and TRM. In the therapeutic setting after HCT, FMT has been used for the treatment of refractory CDI^98–101^ and steroid-refractory acute GVHD (see below). There has been a large variability in approach, as we will discuss in the following sections. A summary of the key features of the previous FMT trials in the field with at least 10 patients treated with FMT is shown in Tables 1–3 ^102–108^

**Table 1.** Study characteristics.

ID	First author	Year	Country	N	Patient population	Age	Prospective	Controls	Randomized
1	Spindelboeck^102^	2014–2019	Austria	10	Steroid-refractory GI aGVHD	54 (24–67)	Yes	No	No
2	Goeser^103^	2017–2019	Germany	11	Steroid-refractory aGVHD	54 (30–76)	No	No	No
3	Battipaglia^104^	2014–2017	France	10	MDRB colonization	48 (16–64)	No	No	No
4	Van Lier^105^	2019	Netherlands	15	Steroid-refractory/dependent GI aGVHD	57 (20–72)	Yes	No	No
5	Taur^111^	2018	USA	14 11 controls	Post-allogeneic HCT (prophylactic) and low fecal Bacteroidetes abundance	55 (28–72)	Yes	Yes	Yes
6	DeFilipp^106^	2018	USA	13 32 controls	Post-allogeneic HCT (prophylactic)	63 (26–71)	Yes	Yes	No
7	Zhao^107^	2018	China	23 18 controls	Grade IV steroid-refractory GI aGVHD	30 (13–55)	Yes	Yes	No
8	Rashidi^108^	2023	USA	49 25 placebo	Post-allogeneic HCT (prophylactic)	54 (15)	Yes	Yes (placebo)	Yes (double-blinded)

aGVHD:acute graft-versus-host disease; GI: gastrointestinal; HCT: hematopoietic cell transplantation; MDRB: Multidrug-resistant bacteria.

**Table 2.** FMT administration details.

ID	FMT time (days from HCT)	Route	FMT material	Dose	Broad-spectrum antibiotics within 2 weeks after FMT	Doses
1	78 (35–487)	Ileocolonoscopy	Frozen	200 ml	5 of 6 non-responders, 0 responders	1–6
2	169 (range 44–642)	NJ tube: 2 Oral capsules: 9	Fresh: 2 Frozen: 9	30–50 capsules/dose	7 patients	1 (*n* = 10) 2 (*n* = 1)
3	Pre-HCT: 28 (9–46), *n* = 4 Post-HCT: 163 (98–344), *n* = 6	Enema: 9 NG tube: 1	Fresh: 2 Frozen: 8	Median 84 g (range: 43–104)	N/A	1 (*n* = 7) 2 (*n* = 3)
4	7 (1.6–49.9) m	ND tube	Fresh	400 ml	+	1
5	18 (8-27)*	Enema	Frozen	N/A	+	1
6	27 (19–45)	Oral capsules	Frozen	38.6 g	Antibacterials stopped at least 2 days before FMT	1
7	N/A	NJ or NG tube	Frozen	40–50 ml	+	1 (*n* = 9) 2 (*n* = 11) 3 (*n* = 2) 6 (*n* = 1)
8	24 (11–63)	Oral capsules	Frozen	5 capsules (≥10^11^ bacteria/capsule)	Antibacterials stopped at least 2 days before FMT	1 (*n* = 58) 2 (*n* = 13) 3 (*n* = 3)

FMT: fecal microbiota transplantation; MDRB: Multidrug-resistant bacteria; N/A: not available; ND: nasoduodenal; NG: nasogastric; NJ: nasojejunal. * : after neutrophil engraftment

**Table 3.** FMT safety and efficacy.

ID	Primary endpoint	Secondary endpoints	Major attributed toxicity	Outcome
1	Response (3 m)	OS, GI aGvHD related survival, recurrence	1 possible pathogen transmission (fatal adenoviremia)	CR/PR: 4 Improved alpha diversity in responders Compositional similarity to FMT donor in responders *Enterococcus* and *Lactobacillus* predicted NR
2	Response (1 m)	_	None	CR/PR: 10 Improved alpha diversity and compositional switch toward donor
3	Decolonization	_	None	Decolonization: 7
4	Response	_	None	CR: 10; NR: 5 Improved alpha diversity in responders Compositional similarity to FMT donor in responders *Bluaita*, *Clostridia*, and predicted butyrate-producing genera increased in responders
5	CDI (1 y)	Systemic bacterial and viral infections	N/A	Restored alpha diversity, composition, and commensal bacteria
6	Feasibility	BSI, CDI, aGVHD, TRM, PFS, OS	Grade 3 abdominal pain: 1	93% feasibility Grade III-IV aGVHD: 2 Bacteremia: 1; CDI: 1 TRM: 1; 1y OS and PFS: 85% Higher alpha diversity after FMT
7	EFS and OS (3 m)	_	None	OS improved; EFS unchanged
8	4-month all-cause infection rate	Grade II-IV aGVHD	None	No significant change in infection rate More grade II-IV acute GVHD after FMT (limited analysis)

Year: year(s) of study unless not reported in which case the article’s publication year is provided. Goeser et al. is the only multi-center study (2 centers) and the only one using pooled donor product (1 patient). Spindelboeck et al. is the only study using the stem cell donor also as the FMT donor (9 patients). All studies were in the allogeneic HCT setting. All studies used allogeneic FMT donors except the study by Taur et al, which used autologous FMT. Age is shown as median (range) except in Rashidi et al. where mean (standard deviation) is reported. Days from HCT are shown as median (range) except in Goeser et al. where mean (range) is reported.

aGVHD: acute graft-versus-host disease; BSI: bloodstream infection; CDI: Clostridioides difficile infection; cGVHD: chronic graft-versus-host disease; CR: Complete response; EFS: Event-free survival time; FMT: fecal microbiota transplantation; GI: gastrointestinal; NR: no response; OS: Overall survival; PR: Partial response; TRM: transplant-related mortality.

### Eradication of antibiotic-resistant bacteria (ARB)

The efficacy of FMT in eradicating highly refractory cases of CDI has inspired its use in HCT recipients (both before and after HCT) with MDRO colonization. The ultimate goal of such attempts would be to prevent clinical infections with these organisms. In the largest report thus far with clinical outcomes,^109^ 11 patients received third-party FMT at least 2 weeks before their planned HCT. Eight of these patients proceeded to allogeneic HCT. A non-randomized comparator group was used. Compared to the 6-month period before FMT, patients receiving FMT had a shorter hospitalization and fewer days on carbapenems during HCT. This change did not occur in the comparator group. In another retrospective study,^110^ 8 MDRO-colonized patients received third-party FMT before HCT. When compared to their pair-matched controls without MDRO colonization, 1-year survival was not different. In contrast, MDRO-colonized patients who did not receive FMT had a significantly lower 1-year survival compared to their matched controls without MDRO organization. These findings suggested that the mortality increment due to MDRO colonization may be ameliorated by FMT. Based on limited available data, successful decolonization seems to occur in 50–70% of MDRO-colonized HCT recipients.^104^

Clinical outcomes after MDRO decolonization may improve, but the evidence is weak. Most studies have been small, retrospective or non-randomized, and without a primary clinical endpoint. With improved supportive care and antibiotics, a randomized trial to demonstrate better survival after FMT vs. placebo in MDRO-colonized patients will require a large sample size which may not be feasible. BSI as a clinical endpoint may be easier to study, and it is an important endpoint because BSIs are associated with re-hospitalization, increased healthcare costs, and more dysbiosis due to antibiotic exposure which could in turn increase the risk of GVHD.

### Restoration of microbiota diversity and commensals

Two randomized trials thus far have evaluated the effect of FMT on the microbiota after allogeneic HCT. In the first trial, 25 allogeneic HCT recipients were randomized after neutrophil engraftment to receive autologous FMT vs. no intervention.^111^ A unique aspect of this study was that only patients with a low fecal abundance of Bacteroidetes were treated. FMT improved microbiota alpha diversity and compositional recovery toward baseline including Lachnospiraceae, Ruminococcaceae, and Bacteroidetes which contain many commensal bacteria. Shotgun metagenomic sequencing suggested enrichment in the control group in antimicrobial-peptide resistance and pathways associated with microbial virulence, biofilm formation, and bacterial flagella assembly. In the second trial,^108^ we randomized 74 patients to receive FMT or placebo (2:1 ratio) after neutrophil engraftment. FMT enhanced recovery of alpha diversity and *Collinsella* and reduced the abundance of expanded genera *Enterococcus a*nd *Dialister*. Notably, *Blautia* recovered to the same extent in both arms, arguing against an essential FMT impact. More details about the design of this trial and clinical outcomes are provided in the next section.

### FMT as a prophylactic intervention to improve transplant outcomes

We recently reported the results of our randomized placebo-controlled FMT trial in allogeneic HCT recipients with 4-month all-cause infection rate as the primary endpoint.^108^ The trial also had an independent cohort of patients with acute myeloid leukemia receiving induction chemotherapy, which is not of primary interest for this review. Five standardized third-party oral capsules were administered per dose. Strict donor and product screening were implemented to minimize the risk of pathogen transmission. The first treatment was given at the time of neutrophil recovery and at least 2 days after discontinuation of antibacterial antibiotics. Patients re-exposed to antibacterial antibiotics after dose 1 received up to 2 more doses. Although infection rate was numerically lower after FMT than placebo, the difference did not reach statistical significance (*P* = 0.49). Acute toxicities were limited to mild gastrointestinal symptoms. The incidence of acute GVHD (secondary endpoint) was higher in the FMT arm, but the small number of events and imbalance in GVHD prophylaxis regimens between the two arms prohibited a firm conclusion. A randomized placebo-controlled study powered to evaluate the effect of FMT on preventing acute GVHD rate is needed.

### FMT as a treatment for refractory acute GVHD

Refractory acute GVHD is a life-threatening complication of HCT in need of novel therapeutics. Current treatment is only partially effective and most patients who are refractory to two lines of treatment die. Standard treatment is based on the use of immunosuppressive medications with a wide range of toxicities and does not address other aspects of GVHD pathogenesis such as tissue repair and tolerance.

In the first use of FMT to treat steroid-refractory acute GVHD,^112^ three patients with steroid-refractory and one with steroid-dependent acute intestinal GVHD received 1–2 sessions of third-party FMT via a nasoduodenal tube (34–307 grams). Three patients achieved a complete response and 1 achieved a partial response, with no major treatment-related toxicity. The 3 complete responders were not exposed to antibacterial antibiotics during the two weeks after FMT, except trimethoprim-sulfamethoxazole in one patient and a brief course of levofloxacin in another. In contrast, the patient with a partial response was heavily exposed to anti-anaerobic antibiotics after FMT. It is tempting to hypothesize that such exposure decreased the efficacy of FMT, but it is also possible that the use of these antibiotics reflected a worse case of GVHD, with less response to FMT even without antibiotic exposure. Effector regulatory T cells increased in the circulation after FMT, suggestive of a systemic anti-inflammatory response. Taxa that increased after FMT in complete responders included *Bacteroides*, *Faecalibacterium*, *Lactobacillus*, and *Bifidobacterium*. *Escherichia* was the dominant taxon after FMT in the partial responder. FMT did not normalize microbiota diversity, suggesting that full restoration of the microbiota may not be necessary for clinical response.

Several reports were subsequently published on the use of FMT in patients with refractory acute GVHD.^102,103,105,107,113^ In the largest prospective study thus far,^107^ 41 patients with steroid-refractory acute gut GVHD received third-party FMT (23 patients). A control group of 18 patients who received other treatments was included in this non-randomized study. In addition to FMT, the FMT cohort received next-line immunosuppressive treatment, thus FMT was an adjunct treatment rather than a replacement for standard immunosuppression. The FMT cohort received 1–6 FMT treatments via a nasojejunal or gastric tube. Response and survival were higher in the FMT cohort, without increased toxicity. The relative abundance of Firmicutes and Bacteroidetes seemed to increase after FMT while the relative abundance of Proteobacteria seemed to decrease, although a robust analysis was not possible due to the small number of stool samples. In another prospective single-arm study,^105^ 15 patients with steroid-refractory or -dependent acute intestinal GVHD safely received one third-party FMT via nasoduodenal infusion 2 hours after a bowel lavage. Prior immunosuppressive therapy was not changed. Ten patients achieved a complete response within a month, associated with signals for improved alpha diversity, partial donor microbiota engraftment, and increased abundance of Clostridiales, *Blautia*, and predicted butyrate-producing taxa. However, confidence intervals were large, likely contributing to non-significant differences between responders and non-responders.

Although a randomized trial of FMT for the treatment of refractory acute GVHD has not been published, the available evidence suggests efficacy. An important question to be addressed is the mechanism by which microbiota restoration may improve acute GVHD outcomes. Factors other than a poorly controlled alloimmune attack seem to be involved in steroid-refractory acute GVHD. Tissue tolerance, a concept adopted from non-transplant settings and recently introduced in the HCT setting, posits that tissue-intrinsic factors may have a key role in both progression and resolution of acute GVHD.^114^ Such factors do not oppose the attacking agents (e.g. T cells) directly, but rather help with tissue resistance and repair. Whether and how the microbiota may contribute to this process is unclear, but ideas such as butyrate serving as a growth factor for injured colonocytes could be entertained.^81,82,115,116^

### Toxicity of FMT

Besides occasional and typically transient gastrointestinal side effects (nausea, bloating, abdominal pain, diarrhea) that tend to happen in the first 1–2 days after FMT, the main risk associated with FMT is pathogen transmission. The introduction of millions of additional bacteria to the gut, particularly in the setting of profound immunosuppression and intestinal barrier damage after HCT can potentially result in microbial translocation to the bloodstream infection and life-threatening sepsis.^117^ The risk is not limited to bacteria. As an example, eosinophilic gastroenteritis due to Norovirus transmitted via FMT in an allogeneic HCT patient has been reported.^118^ Major regulatory organizations have established strict rules for screening FMT donors and their stool. It is noteworthy that a BSI after FMT does not necessarily point to the FMT product as the origin of infection. Strain-level analysis of 13 BSI events after FMT in patients with steroid-refractory or -dependent acute GVHD did not find the BSI-associated strains in the FMT product.^119^ Several bacteria isolated from blood cultures (*Enterococcus faecium*, *Escherichia coli*, *Pseudomonas aeruginosa*, and *Acinetobacter baumannii*) were present in stools sample before BSI, suggesting that the patients’ own microbiota was likely the source of infection. In our randomized trial, we found no bloodstream infections attributable to FMT.^108^

An arbitrary interval after neutrophil engraftment has been commonly used to administer FMT after HCT, an approach based on the perceived increased risk of infection in a neutropenic patient. However, no compelling evidence indicates the pre-engraftment period as an especially high-risk period for FMT. As pre-engraftment HCT patients are severely immunosuppressed and at high risk for infection even without FMT, it is important to perform FMT in such patients only in the context of clinical trials, and ideally in a randomized setting to permit distinguishing FMT-related safety signals from baseline risks.

## Concluding remarks

### What are the lessons learned?

Several associations have been reported between intestinal dysbiosis and poor clinical outcomes after HCT and cellular therapy. The evidence is currently stronger in the HCT setting, but recent studies have found similar associations after CAR-T therapy. In particular, early loss of obligate anaerobic commensal bacteria and expansion of *Enterococcus* and mucolytic species may potentiate complications and lead to poor outcomes. The multitude of factors mediating both clinical outcomes and gut microbiota in these settings makes causal links difficult to establish in humans. Controlled murine experiments have suggested mechanistic connections, but generalization to the human setting is not trivial. In particular, procedures to access the mucosal-adherent microbiota in this patient population are risky and stool samples represent luminal microbiota, which is different from the likely more relevant mucosal-adherent microbiota. One approach to demonstrate a causal link in humans is by randomized trials. Randomization reduces the imbalance between the arms in measured and unmeasured variables. Different outcomes between the arms in such a design would strongly suggest causality. The only completed randomized controlled FMT trial after allogeneic HCT with primary (infections) and secondary (acute GVHD) clinical endpoints did not improve outcomes despite substantial amelioration of dysbiosis.^108^

### What is the path forward?

Novel aspects of microbiota and its role in tissue homeostasis need to be translated to clinical trials. Although most attention has been paid to the bacteria in the setting of FMT, the possible role of other microorganisms such as viruses (e.g. phages) is becoming more clear. Removing the bacterial component of donor stool by sterile filtration maintains FMT efficacy on gut microbiota composition and host metabolome.^120^ Sterile fecal filtrates contain bacterial debris, proteins, antimicrobial compounds, metabolic products, oligonucleotides/DNA, and viruses, but not intact bacteria. Such filtrates (a.k.a. fecal virome transplantation) have been tested with success for the treatment of recurrent CDI in small series^121^ and may have a role in HCT recipients too. Currently, there are no published FMT trials in CAR-T recipients, and to our knowledge, no such trial is ongoing in this setting. Given recent findings about microbiota associations with CAR-T efficacy and toxicity, FMT should be tested in clinical trials in this setting. The interval between CAR-T referral and lymphodepletion chemotherapy is often several weeks long, providing sufficient opportunity for FMT to modulate the microbiota in patients deemed high-risk based on the available associational data. If the association between microbiota injury and disease progression after CAR-T therapy is confirmed in more cohorts, pre- or post-CAR FMT may be a testable strategy to boost treatment effect and improve cure rates.

### What are the open questions that need to be addressed before FMT becomes a standard therapeutic regimen?

From a mechanistic standpoint, the determinants of donor microbiota engraftment in the HCT setting are unknown. In addition, the effect of FMT on non-bacterial compartments of the microbiome (e.g. virome, mycobiome) is rather unexplored.^122^

The optimal timing and schedule of administration and minimum required dose of FMT are unknown. With rigorous screening of donors and stools to minimize the risk of pathogen transmission, the existence of a specific neutrophil count threshold for FMT is questionable. Careful examination of FMT safety during severe neutropenia in a properly designed clinical trial with safety as the primary endpoint would be informative as the pre-engraftment period when antibiotic pressure tends to be maximal may offer a new window of opportunity for intervention on the microbiota. Most studies have administered FMT on a single day or on 2 consecutive days. Whether a more sustained schedule extending for several days and potentially delivering a larger dose of microbiota is beneficial is unknown. The route of administration is another unknown factor potentially influencing efficacy. Besides the oral encapsulated form,^103,106,108,113^ FMT has been administered by enema,^104,111,123^ via nasogastric/nasoduodenal tube,^104,105,109,112,124^ or by upper^127^ or lower endoscopy.^102^ Another open question concerns the FMT source. Both third-party^102,104,105,108,109,124,125^ and autologous^111^ products have been used. While an autologous product may be simpler and less expensive to manufacture, most HCT recipients have had microbiota insults before referral for transplant and the efficacy of a product manufactured from a “scarred” microbiota needs further investigation. Similarly, whether a single “super donor”,^126^ several donors (one used for each patient), or pooled donors provide better outcomes is unknown. Pooling stool from multiple donors may generate a more diverse microbiota in the product, but the value of an extra-high diversity beyond what an average healthy individual has is unknown. Whether concurrent use of antibiotics may be detrimental to FMT efficacy and safety is also unclear. Related to this point, the optimal washout after the last antibiotic use has not been established. Finally, the efficacy of “microbiota conditioning” by antibiotic gut decontamination before FMT to increase donor microbiota engraftment has not been determined.
